# A phase I dose-escalation study of selumetinib in combination with docetaxel or dacarbazine in patients with advanced solid tumors

**DOI:** 10.1186/s12885-017-3143-6

**Published:** 2017-03-06

**Authors:** Patricia M. LoRusso, Jeffrey R. Infante, Kevin B. Kim, Howard A. Burris, Gregory Curt, Ugochi Emeribe, Delyth Clemett, Helen K. Tomkinson, Roger B. Cohen

**Affiliations:** 1 Yale Cancer Center, PO Box 208028, New Haven, CT 06520-8028 USA; 2 Sarah Cannon Research Institute, Nashville, TN USA; 3 Tennessee Oncology PLLC, Nashville, TN USA; 4 California Pacific Medical Center, San Francisco, CA USA; 5 AstraZeneca, Wilmington, DE USA; 6 AstraZeneca, Macclesfield, UK; 7 Fox Chase Cancer Center, Philadelphia, PA USA

**Keywords:** Selumetinib, Dose-escalation, Advanced solid tumors, Docetaxel, Dacarbazine

## Abstract

**Background:**

The RAS/RAF/MEK/ERK pathway is constitutively activated in many cancers. Selumetinib (AZD6244, ARRY-142886) is an oral, potent and highly selective, allosteric MEK1/2 inhibitor with a short half-life that has shown clinical activity as monotherapy in phase I and II studies of advanced cancer. Preclinical data suggest that selumetinib may enhance the activity of chemotherapeutic agents. We assessed the safety, tolerability, and pharmacokinetics (PK) of selumetinib (AZD6244, ARRY-142886) in combination with docetaxel or dacarbazine in patients with advanced solid tumors.

**Methods:**

This study was a phase I, open-label, multicenter study in patients aged ≥18 years with advanced solid tumors who were candidates for docetaxel or dacarbazine treatment. Part A of the study (dose escalation) evaluated safety, tolerability, PK, and maximum tolerated dose (MTD) of selumetinib twice daily (BID) with docetaxel 75 mg/m^2^ or dacarbazine 1000 mg/m^2^ administered every 21 days. Patients receiving docetaxel could be administered primary prophylactic granulocyte-colony stimulating factor according to standard guidelines. Part B of the study (dose expansion) further evaluated safety, tolerability, and PK in 12 additional patients at the MTD combinations determined in part A.

**Results:**

A total of 35 patients received selumetinib plus docetaxel, and 25 received selumetinib plus dacarbazine. The MTD of selumetinib was 75 mg BID in combination with either docetaxel (two dose-limiting toxicity [DLT] events: neutropenia with fever, and thrombocytopenia) or dacarbazine (one DLT event: thrombocytopenia). Common adverse events occurring with each treatment combination were diarrhea, peripheral/periorbital edema, fatigue, and nausea. PK parameters for selumetinib and docetaxel or dacarbazine were similar when administered alone or in combination. Partial responses were reported in 6/35 patients receiving selumetinib plus docetaxel and 4/25 patients receiving selumetinib plus dacarbazine.

**Conclusions:**

The combinations of selumetinib plus docetaxel and selumetinib plus dacarbazine demonstrated manageable safety and tolerability profiles and preliminary signs of clinical activity in patients with advanced solid tumors.

**Trial registration:**

ClinicalTrials.gov NCT00600496; registered 8 July 2009.

**Electronic supplementary material:**

The online version of this article (doi:10.1186/s12885-017-3143-6) contains supplementary material, which is available to authorized users.

## Background

The intracellular RAS/RAF/MEK/ERK pathway converges on MEK1/2, of which the only known substrates are ERK1/2. Constitutive activation of the pathway is implicated in cell proliferation and is central to driving cancer growth and progression [1, 2]. The integral role of MEK1/2 in this signaling cascade highlights its potential as a therapeutic target. Selumetinib (AZD6244, ARRY-142886) is an oral, potent, and highly selective, allosteric MEK1/2 inhibitor [3] with a short half-life [4, 5]. Clinical activity of selumetinib monotherapy in some patients with advanced solid tumors has been reported in phase I [4, 6] and phase II studies [7–10], and the recommended dose for selumetinib monotherapy is 75 mg twice daily (BID). The potential benefit of combining MEK inhibitors with chemotherapy has been demonstrated in preclinical studies of tumor xenograft models, in which selumetinib in combination with cytotoxic agents, such as docetaxel or the dacarbazine derivative temozolomide, showed enhanced tumor growth inhibition compared with selumetinib monotherapy, or chemotherapy alone [1, 11]. Selumetinib plus docetaxel in previously treated patients with *KRAS*-mutant advanced non-small cell lung cancer (NSCLC) [12], and selumetinib plus dacarbazine in patients with *BRAF*-mutant metastatic melanoma [13], have more recently been assessed in phase II trials. Additional phase II and phase III trials of selumetinib plus docetaxel in advanced NSCLC were also initiated (ClinicalTrials.gov identifiers: NCT01750281; NCT01933932). Here we present the phase I study on which development of these combinations was based.

The objectives of this four-arm dose-escalation study were to assess the safety, tolerability, pharmacokinetics (PK), and maximum tolerated dose (MTD) of selumetinib in combination with selected anticancer therapies (docetaxel, dacarbazine, erlotinib, or temsirolimus) in patients with advanced solid tumors. An exploratory assessment of tumor response was also conducted. A single-institution assessment of patients with metastatic melanoma enrolled in this study has previously been reported [14]. In consideration of the notable differences in safety and tolerability profiles when combining selumetinib with different classes of cancer therapeutics, we present here data from 60 patients who received selumetinib in combination with cytotoxic agents (docetaxel or dacarbazine). Results for those patients who received selumetinib in combination with other molecularly targeted therapies (erlotinib or temsirolimus) are presented by Infante et al. in a companion manuscript (in preparation).

## Methods

This phase I, open-label, multicenter, dose-escalation study (NCT00600496) was conducted at four centers in the USA between December 2007 and August 2010 (data cut-off occurring 6 months after the last patient began treatment).

### Patient selection

Patients with advanced solid tumors who would be candidates for docetaxel or dacarbazine treatment as a standard of care, or those who might have derived benefit from combination therapies with these agents, were eligible for the study. Other eligibility criteria included: age ≥18 years; measurable and/or non-measurable disease lacking curative options; World Health Organization (WHO) performance status 0 or 1; and calculated serum creatinine clearance >50 mL/min.

Patients meeting any of the following criteria were excluded from the study: prior treatment with a MEK inhibitor; received an investigational drug within 30 days of entering the study, and/or had not recovered to < grade 1 toxicity; received radiotherapy or standard chemotherapy within 21 days of study entry; use of strong cytochrome (CYP)1A2 or 3A4 inducers and/or inhibitors; brain metastases or spinal cord compression unless treated and stable (>1 month) and off steroids; having factors that increased the risk of QT prolongation or arrhythmic events or QTc interval of >450 ms for males or >470 ms for females; or inadequate bone marrow, hepatic, cardiac, or renal function.

All patients provided written informed consent and the study was conducted in accordance with Good Clinical Practice guidelines and the Declaration of Helsinki. The protocol was approved by the institutional review board at each study site (Additional file 1: Table S1).

### Study design and dosing

For each treatment arm, the study was conducted in two parts: part A (dose escalation) enrolled cohorts of three to six evaluable patients and assessed the safety, tolerability, PK, and MTD of selumetinib in combination with either docetaxel or dacarbazine; part B (dose expansion) further evaluated the safety, tolerability, and PK in a minimum of 12 additional patients at the MTDs for combination treatments determined in part A. The study safety review committee (SRC), comprising representatives from the study sponsor and at least one investigator, assessed the available safety and PK data. Dose-limiting toxicities (DLTs) in the study were defined as those related to treatment and occurring within the first 28 days of therapy. Hematologic DLTs were defined as afebrile grade 4 neutropenia for >5 days, grade 4 neutropenia associated with fever, or grade 4 thrombocytopenia. Non-hematological DLTs were defined as any ≥ grade 3 adverse event (AE) for >7 days that could not be controlled to grade ≤2 with appropriate treatment.

Patients received intravenous docetaxel 75 mg/m^2^ or dacarbazine 1000 mg/m^2^ over 60 mins on day 1 of each 21-day cycle. Selumetinib was initiated BID, orally, beginning on day 3 of cycle 1. Patients could remain on combination treatment or selumetinib monotherapy after discontinuation of chemotherapy, providing they were continuing to derive clinical benefit, until disease progression or intolerable AEs occurred. Patients receiving docetaxel could be administered primary prophylactic granulocyte-colony stimulating factor (ppG-CSF), including pegylated G-CSF, according to standard guidelines.

Dose exploration commenced at the starting dose level of selumetinib 50 mg BID. Patients were enrolled into part A in initial cohorts of three to six patients and subsequent dose levels were determined by the SRC, which reviewed the emerging tolerability and safety profile on an ongoing basis, and upon completion of each dose level cohort. In addition, the predicted exposure to selumetinib at each dose level evaluated was not to exceed the exposures previously observed at the monotherapy MTD of 75 mg BID [4]. Patients were considered evaluable if they had received at least 28 days of therapy from cycle 1/day 1, received approximately 80% of the planned doses of selumetinib, had experienced a DLT, or at the discretion of the SRC. The combination MTD in this study was defined as the highest selumetinib dose achieved at which no more than one of six evaluable patients experienced a DLT. In part B (dose expansion) of the study, an additional 12 evaluable patients received treatment at the combination MTDs.

### Assessments

#### Tolerability

Safety assessments included: all AEs graded using National Cancer Institute Common Terminology Criteria for Adverse Events (CTCAE) version 3.0; vital signs (including blood pressure, pulse rate, weight, and body temperature); electrocardiogram; Multi-Gated Acquisition (MUGA) scan; clinical chemistry; brain natriuretic peptide; troponin I; hematology; urinalysis; and ophthalmologic examinations. Incidence of DLTs was also recorded.

#### Pharmacokinetics assessments

Pharmacokinetic parameters of selumetinib, N-desmethyl selumetinib, docetaxel, and dacarbazine were determined following administration of each drug alone and in combination. Blood sampling was performed pre-dose and after chemotherapy infusion as follows: cycle 1/day 1 (selumetinib) and cycle 2/day 1 (combination) for measurement of docetaxel or dacarbazine levels; and cycle 1/day 3 (selumetinib) and cycle 2/day 1 (combination).

Maximum plasma concentration (C_max_) and time to reach the C_max_ (T_max_) were determined on day 1, 3, and 22 of cycle 1 for docetaxel or dacarbazine, and day 3 and 22 of cycle 1 for selumetinib. Area under the plasma concentration-time curve from 0 to 12 h post dose (AUC_(0–12)_) was calculated using the linear trapezoidal rule. When more than one maximum occurred, the reported value was assigned to the first occurrence.

#### Tumor response

Objective tumor response was assessed according to Response Evaluation Criteria In Solid Tumours (RECIST) (version 1.0) with baseline tumor assessments up to 4 weeks before the planned first dose of selumetinib. Subsequent tumor assessments were conducted prior to the third cycle, every alternate cycle thereafter, and on withdrawal of treatment.

### EGFR and KRAS mutation analyses

Mutation status was an exploratory endpoint and an optional part of the protocol. DNA was extracted from formalin-fixed paraffin-embedded tissue samples using the Cobas™ DNA Sample Preparation kit (Roche Molecular Systems Inc., Pleasanton, CA, USA). Plasma DNA was extracted using a non-commercial plasma preparation kit supplied by Roche. DNA was assayed using the Cobas™ KRAS Mutation Test and Cobas™ EGFR Mutation Test (Roche) according to the manufacturer’s protocols. The *EGFR* mutation assay covered the following mutations: exon 18 G719X (G719A, G719C, and G719S); exon 19 deletions and complex mutations; exon 20 S768I, T790M, and insertions; and exon 21 L858R. The *KRAS* mutation assay covered mutations in codons 12, 13 (exon 2), and 61 (exon 3). Data were generated and analysed using the Cobas z480.

### Statistical analysis

Three populations were defined for analysis: safety population, evaluable for dose escalation (part A), and evaluable for PK analysis. The safety population included all patients who received one or more doses of selumetinib and chemotherapy. Patients were considered evaluable for dose escalation if they had received approximately 80% of the planned doses of selumetinib in cycle 1, provided PK data, and had all safety evaluations performed or experienced a DLT, or at the discretion of the SRC. Patients who could not complete 80% of planned doses or had to drop out due to toxicity were considered DLT evaluable. The evaluable for PK analysis population included all patients with concentration-time data available.

No formal statistical hypothesis testing was performed on the data; all data reported are based on summary statistics: frequency counts and percentages for categorical data and mean, median, range, standard deviation, geometric mean, and % co-efficient of variation for continuous data as appropriate.

## Results

### Patient characteristics and disposition

**Fig. 1:**
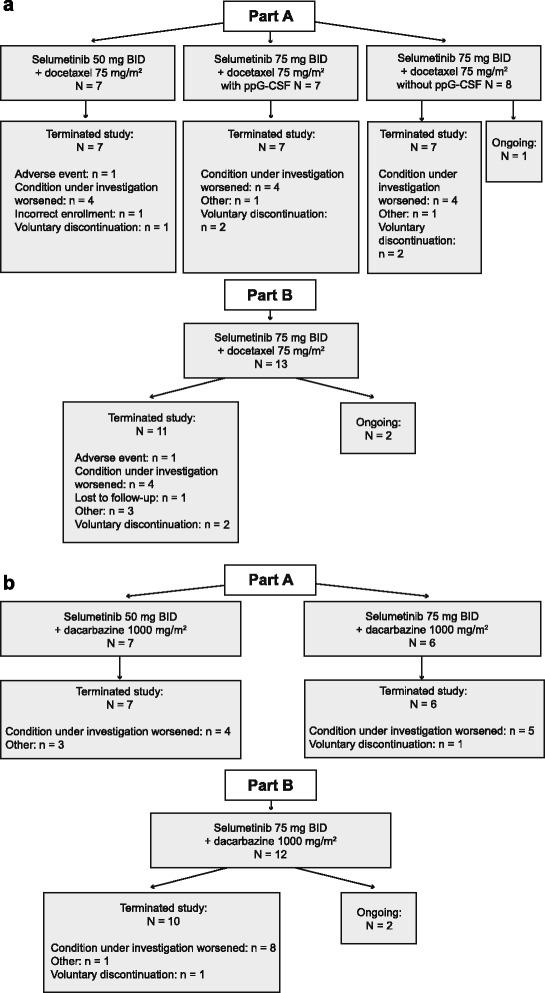
Patient disposition at the time of data cut-off for primary safety analysis (August 2010) in the (**a**) selumetinib plus docetaxel and (**b**) selumetinib plus dacarbazine treatment arms. BID, twice daily; ppG-CSF, primary prophylactic granulocyte-colony stimulating factor

A total of 35 patients received selumetinib plus docetaxel and 25 patients received selumetinib plus dacarbazine. The disposition of patients receiving selumetinib plus docetaxel or selumetinib plus dacarbazine is summarized in Fig. 1.

Among patients who received selumetinib plus docetaxel, 22 were treated in the dose-escalation phase (part A); seven patients received selumetinib 50 mg BID plus docetaxel and 15 patients received selumetinib 75 mg BID plus docetaxel. Thirteen patients were included in the dose expansion phase (part B), and received selumetinib 75 mg BID plus docetaxel. One patient from part A and two from part B (all receiving selumetinib 75 mg BID) remained on treatment at the time of the data cut-off (August 2010).

Thirteen patients were treated with selumetinib plus dacarbazine in the dose-escalation phase (part A); seven patients received selumetinib 50 mg BID plus dacarbazine and six patients received selumetinib 75 mg BID plus dacarbazine. Twelve patients were included in the dose-expansion phase (part B), receiving selumetinib 75 mg BID plus dacarbazine. Two patients, both from part B, remained on treatment at the time of the data cut-off.

Baseline characteristics in the overall study population were typical of patients with advanced solid tumors (Table 1).

**Table 1:** Baseline characteristics of patients

Characteristics, *n* (%)	Selumetinib + docetaxel (*N* = 35)	Selumetinib + dacarbazine (*N* = 25)
Age, years; mean (SD)	58.9 (9.5)	57.1 (11.5)
Male	21 (60.0)	15 (60.0)
Female	14 (40.0)	10 (40.0)
Race		
White	33 (94.3)	25 (100.0)
Black/African American	2 (5.7)	0
WHO performance status		
0	14 (40.0)	12 (48.0)
1	21 (60.0)	13 (52.0)
Primary tumor site		
Skin/soft tissue	11 (31.4)	17 (68.0)
Lung	8 (22.9)	3 (12.0)
Breast	3 (8.6)	0
Colorectal	2 (5.7)	0
Esophagus	2 (5.7)	0
Ovary	2 (5.7)	0
Head and neck	1 (2.9)	1 (4.0)
Uveal	1 (2.9)	1 (4.0)
Other^a^	5 (14.3)	3 (12.0)
Mean prior systemic treatments	3.1	2.0
Prior therapy, *n* (%)		
Chemotherapy	30 (85.7)	18 (72.0)
Platinum compounds	24 (68.6)	9 (36.0)
Taxanes	18 (51.4)	8 (32.0)
Pyrimidine analogues	6 (17.1)	1 (4.0)
Anthracyclines	5 (14.3)	3 (12.0)
Radiotherapy	18 (51.4)	13 (52.0)
Other systemic anticancer therapy^b^	8 (22.9)	8 (32.0)
Immuno/hormonal therapy	6 (17.1)	0
Hormonal therapy	3 (8.6)	0
Immunotherapy	2 (5.7)	10 (40.0)
Prior lines of chemotherapy, *n* (%)		
0 or 1	17 (48.6)	21 (84.0)
2 or 3	10 (28.6)	2 (8.0)
4+	8 (22.9)	2 (8.0)

^a^Includes bladder, lymph nodes, melanoma, muscle, prostate, pancreas, stomach, unknown primary, and uterus

^b^Includes monoclonal antibodies, vaccines, small molecule targeted agents, and investigational drugs

SD, standard deviation; WHO, World Health Organization

### Selumetinib in combination with docetaxel

#### Dose-limiting toxicities

In part A, there were no DLTs in patients receiving selumetinib 50 mg BID plus docetaxel. As local differences in the use of ppG-CSF emerged during the study, patients treated with selumetinib 75 mg BID plus docetaxel were stratified into two subgroups for analysis: patients who did or did not receive ppG-CSF during cycle 1. There were no DLTs in seven patients treated with selumetinib 75 mg BID plus docetaxel with ppG-CSF. Two of eight patients receiving selumetinib 75 mg BID plus docetaxel without ppG-CSF experienced DLTs (grade 4 febrile neutropenia and grade 4 thrombocytopenia) (Table 2). Selumetinib 75 mg BID plus docetaxel with ppG-CSF was defined as the MTD in part A.

**Table 2:** Summary of cohorts and dose escalation based on dose-limiting toxicity

	Part	Selumetinib dose	*n* (evaluable for dose escalation)	Evaluable patients with a DLT	DLT information
Selumetinib in combination with docetaxel					
	A	50 mg BID	7 (6)	0	NA
		75 mg BID with ppG-CSF	7 (6)	0	NA
		75 mg BID without ppG-CSF	8 (5)	2	Grade 4 neutropenia with fever (*n* = 1) Grade 4 thrombocytopenia (*n* = 1)
	B	75 mg BID with ppG-CSF	12 (NA)	NA	NA
		75 mg BID without ppG-CSF	1 (NA)	NA	NA
Selumetinib in combination with dacarbazine					
	A	50 mg BID	7 (6)	1	Grade 4 thrombocytopenia (*n* = 1)
		75 mg BID	6 (6)	0	NA
	B	75 mg BID	12 (NA)	NA	NA

BID, twice daily; DLT, dose-limiting toxicity; NA, not applicable; ppG-CSF, primary prophylactic granulocyte-colony stimulating factor

In part A, it became apparent that patients treated with selumetinib 75 mg BID plus docetaxel with (*n* = 7) or without (*n* = 8) ppG-CSF had been heavily pretreated (median of five or seven prior systemic therapies, respectively). The SRC therefore recommended that part B (dose expansion) should evaluate a patient population similar to that planned in phase II studies and explore the tolerability of administering selumetinib 75 mg BID plus docetaxel without ppG-CSF. Thus, the part B expansion cohort enrolled less heavily pretreated patients (median of 2.5 [range 1–7] prior systemic therapies) who received selumetinib 75 mg BID plus docetaxel without ppG-CSF, but with G-CSF allowed from cycle 2 onwards. Febrile neutropenia and grade 4 neutropenia occurred in two of 13 patients in part B. The combination of selumetinib 75 mg BID plus docetaxel without ppG-CSF was considered to be tolerable in this less heavily treated patient population.

#### Exposure

Patients in part B received the MTD of selumetinib 75 mg BID plus docetaxel for a median duration of approximately 5 months and 54% of these patients received six or more (21-day) cycles of docetaxel. Long-term follow-up reported that as of June 2015, two patients with skin/soft tissue tumors had received 12 months of combination treatment, and were continuing to receive selumetinib monotherapy at reduced doses (one at 50 mg BID; one at 25 mg BID), both >6 years after first receiving selumetinib. The remaining patient who was still receiving study treatment at data cut-off was withdrawn from the study in September 2010.

#### Tolerability

Diarrhea, peripheral edema, fatigue, nausea, vomiting, rash, and neutropenia were the most commonly reported AEs among patients receiving selumetinib plus docetaxel (Table 3). AEs had a similar frequency across the dose cohorts and were mostly grade 1 or 2. The most commonly reported grade ≥3 AEs across all dose groups were neutropenia (15/35 patients, 43%), fatigue (7/35, 20%), and febrile neutropenia (5/35, 14%). Grade 4 events of neutropenia (>5 days) and febrile neutropenia were reported in 11/28 (39%) and 2/28 (7%) patients receiving selumetinib 75 mg BID plus docetaxel, respectively. Grade 5 AEs of pneumonia and febrile neutropenia were reported in a patient with esophageal cancer while receiving post-progression chemotherapy with FOLFIRI; neither AE was considered by the investigator to be related to study treatment.

**Table 3:** Adverse events: selumetinib in combination with docetaxel or dacarbazine

	Part A			Part B	Part A + B
	Selumetinib 50 mg BID (*N* = 7)	Selumetinib 75 mg BID with ppG-CSF (*N* = 7)	Selumetinib 75 mg BID without ppG-CSF (*N* = 8)	Selumetinib 75 mg BID^a^ (*N* = 13)	Selumetinib 75 mg BID (*N* = 28)
Selumetinib in combination with docetaxel AE category, *n* (%)					
Any AE	7 (100.0)	7 (100.0)	8 (100.0)	12 (92.3)	27 (96.4)
Any grade ≥3 AE	5 (71.4)	1 (14.3)	7 (87.5)	11 (84.6)	19 (67.9)
Any SAE	4 (57.1)	2 (28.6)	5 (62.5)	7 (53.8)	14 (50.0)
Any AE leading to discontinuation	0	0	0	2 (15.4)	2 (7.1)
Most frequently reported AEs (≥20% of patients receiving selumetinib 75 mg BID + docetaxel), *n* (%)					
Diarrhea	5 (71.4)	3 (42.9)	7 (87.5)	11 (84.6)	21 (75.0)
Edema peripheral	5 (71.4)	3 (42.9)	7 (87.5)	10 (76.9)	20 (71.4)
Fatigue	7 (100.0)	2 (28.6)	5 (62.5)	9 (69.2)	16 (57.1)
Nausea	2 (28.6)	3 (42.9)	4 (50.0)	8 (61.5)	15 (53.6)
Vomiting	4 (57.1)	3 (42.9)	4 (50.0)	5 (38.5)	12 (42.9)
Dermatitis acneiform	3 (42.9)	4 (57.1)	3 (37.5)	5 (38.5)	12 (42.9)
Neutropenia	4 (57.1)	1 (14.3)	5 (62.5)	5 (38.5)	11 (39.3)
Constipation	3 (42.9)	2 (28.6)	3 (37.5)	4 (30.8)	9 (32.1)
Dyspnea exertional	3 (42.9)	1 (14.3)	2 (25.0)	5 (38.5)	8 (28.6)
Epistaxis	3 (42.9)	2 (28.6)	2 (25.0)	4 (30.8)	8 (28.6)
Dysgeusia	2 (28.6)	2 (28.6)	1 (12.5)	5 (38.5)	8 (28.6)
Neuropathy peripheral	2 (28.6)	2 (28.6)	3 (37.5)	3 (23.1)	8 (28.6)
Mucosal inflammation	5 (71.4)	3 (42.9)	1 (12.5)	2 (15.4)	6 (21.4)
Nail disorder	1 (14.3)	1 (14.3)	3 (37.5)	2 (15.4)	6 (21.4)
Selumetinib in combination with dacarbazine AE category, *n* (%)					
Any AE	7 (100.0)	6 (100.0)		12 (100.0)	18 (100.0)
Any grade ≥3	7 (100.0)	2 (33.3)		9 (75.0)	11 (61.1)
Any SAE	4 (57.1)	2 (33.3)		4 (33.3)	6 (33.3)
Any AE leading to discontinuation	0	0		1 (8.3)	1 (5.6)
Most frequently reported AEs (≥30% of patients receiving selumetinib 75 mg BID + dacarbazine), *n* (%)					
Diarrhea	4 (57.1)	6 (100.0)		9 (75.0)	15 (83.3)
Nausea	5 (71.4)	5 (83.3)		10 (83.3)	15 (83.3)
Fatigue	2 (28.6)	3 (50.0)		11 (91.7)	14 (77.8)
Decreased appetite	1 (14.3)	3 (50.0)		8 (66.7)	11 (61.1)
Dizziness	1 (14.3)	2 (33.3)		8 (66.7)	10 (55.6)
Headache	1 (14.3)	4 (66.7)		6 (50.0)	10 (55.6)
Vomiting	1 (14.3)	3 (50.0)		7 (58.3)	10 (55.6)
Constipation	3 (42.9)	3 (50.0)		6 (50.0)	9 (50.0)
Dermatitis acneiform	1 (14.3)	2 (33.3)		7 (58.3)	9 (50.0)
Edema peripheral	3 (42.9)	1 (16.7)		8 (66.7)	9 (50.0)
Dyspnea exertional	1 (14.3)	2 (33.3)		6 (50.0)	8 (44.4)
Asthenia	0	2 (33.3)		5 (41.7)	7 (38.9)
Vision blurred	0	1 (16.7)		6 (50.0)	7 (38.9)
Periorbital edema	1 (14.3)	0		6 (50.0)	6 (33.3)

Docetaxel, 75 mg/m^2^; dacarbazine 1000 mg/m^2^

^a^ppG-CSF allowed from cycle 2 onwards

AE, adverse event; BID, twice daily; ppG-CSF, primary prophylactic granulocyte-colony stimulating factor; SAE, serious adverse event

Six patients (17%) experienced AEs relating to vision disturbance, all of which were grade 1. Five patients (14%) reported blurred vision (7 events in total) and 1 patient (3%) receiving selumetinib 75 mg BID without ppG-CSF experienced reduced visual acuity. These AEs are known to have resolved on continuing selumetinib for 5 of the 6 patients, with resolution in the remaining patient unknown. One event of blurred vision was considered related to selumetinib, and one related to selumetinib and docetaxel. AEs related to cardiac disorders were reported in two patients who received selumetinib 75 mg BID: one patient had grade 1 first degree atrioventricular block and another patient had grade 2 cardiac valve disease which was identified 5 days after an SAE of pulmonary hypertension. There were no AEs related to left ventricular ejection fraction (LVEF).

In part A of this study, the incidences of grade ≥3 neutropenia and grade ≥3 febrile neutropenia in patients who received selumetinib 75 mg BID plus docetaxel were lower in those who received ppG-CSF (1/7 patients, 14% and 0%, respectively) compared with those who did not receive ppG-CSF (5/8, 63% and 3/8, 38%, respectively). With selumetinib 75 mg BID plus docetaxel, one patient (14.3%) also receiving ppG-CSF required a dose reduction of selumetinib, whereas three patients who did not receive ppG-CSF required at least one dose reduction of selumetinib. In part B, six patients who received selumetinib 75 mg plus docetaxel without ppG-CSF required a selumetinib dose reduction.

#### Pharmacokinetics analysis

Pharmacokinetic parameters of selumetinib, N-desmethyl selumetinib, and docetaxel (summarized in Table 4) were similar when administered alone and in combination. Plasma concentration time profiles of selumetinib, N-desmethyl selumetinib, and docetaxel were also similar when administered alone or in combination (Additional file 2: Figure S1).

**Table 4:** Pharmacokinetic parameters of selumetinib following dosing alone and in combination with either docetaxel or dacarbazine

	Geometric mean (% co-efficient of variation) [*n* evaluable]							
	Selumetinib + docetaxel				Selumetinib + dacarbazine			
Parameter	Dosed alone		Dosed in combination		Dosed alone		Dosed in combination	
	Selumetinib 50 mg (*N* = 7)	Selumetinib 75 mg (*N* = 19)	Selumetinib 50 mg (*N* = 7)	Selumetinib 75 mg (*N* = 16)	Selumetinib 50 mg (*N* = 7)	Selumetinib 75 mg (*N* = 18)	Selumetinib 50 mg (*N* = 7)	Selumetinib 75 mg (*N* = 15)
Selumetinib								
C_max_, ng/mL	382.5 (114.50)	1165 (62.27)	576.6 (41.39)	1215 (70.09) [13]	933.5 (43.38)	1537 (45.53)	692.7 (56.34)	1343 (74.40)
T_max_, h^a^	4.0 (1.0–12.0)	1.0 (1.0–2.0)	1.53 (0.5–4.0)	1.5 (0.5–4.0) [13]	1.0 (1.0–3.0)	1.0 (0.5–4.0)	1.0 (0.5–1.5)	1.5 (1.0–4.0)
AUC_(0–8)_, ng•h/mL	1144 (52.30)	2735 (43.05)	1788 (25.59)	3788 (57.53) [13]	2351 (23.14)	3784 (31.17)	2460 (44.05)	4544 (45.42)
AUC_(0–12)_, ng•h/mL	1479 (45.02)	2999 (42.33)	2056 (30.40) [5]	5239 (31.04) [9]	2652 (22.38)	4210 (31.55)	2822 (42.48)	5317 (43.00)
N-desmethyl selumetinib								
C_max_, ng/mL	31.79 (60.02)	69.63 (49.65)	29.41 (175.6)	40.91 (52.07) [13]	46.99 (77.29)	71.21 (69.68)	14.89 (161.60)	24.94 (96.48)
T_max_, h^a^	4.0 (1.0–12.0)	1.25 (1.0–4.0)	2.0 (1.0–4.0)	1.5 (0.5–4.0) [13]	1.0 (1.0–2.0)	1.5 (1.0–4.0)	1.5 (0.5–2.0)	2.0 (1.0–4.0)
AUC_(0–8)_, ng•h/mL	120.0 (33.99) [6]	209.4 (37.13)	170.8 (49.61) [5]	150.3 (35.51) [13]	163.8 (70.89)	212.8 (48.59)	102.2 (94.56) [5]	101.5 (72.76)
AUC_(0–12)_, ng•h/mL	151.2 (37.44) [6]	236.5 (36.06)	258.5 (42.52) [3]	172.7 (38.09) [10]	196.0 (70.11)	261.3 (40.61) [17]	277.9 (2.08) [2]	146.4 (58.26) [12]
Docetaxel								
C_max_, ng/mL	2826 (44.32)	2109 (83.65) [15]	2789 (16.66)	2623 (24.33) [13]				
T_max_, h^a^	1.0 (0.5–1.0)	0.5 (0.5–1.5) [15]	0.5 (0.5–1.0)	1.0 (0.5–1.5) [13]				
AUC_(0–8)_, ng•h/mL	3396 (48.98)	2445 (58.67) [15]	2978 (7.75)	2834 (22.27) [13]				
AUC_(0–12)_, ng•h/mL	3493 (49.31)	2533 (55.74) [15]	3049 (8.90) [6]	2871 (25.19) [9]				
Dacarbazine								
C_max_, ng/mL					29.43 (49.76)	30.17 (20.38) [16]	28.86 (34.66)	30.60 (17.87) [18]
T_max_, h^a^					1.0 (1.0–1.5)	1.0 (1.0–1.5)	1.0 (1.0–1.0)	1.0 (1.0–1.5)
AUC_(0–8)_, ng•h/mL					95.92 (39.61) [6]	73.73 (22.24) [15]	83.20 (45.68)	78.82 (24.23) [18]
AUC_(0–12)_, ng•h/mL					144.9 (58.46) [4]	101.5 (23.28) [9]	132.6 (62.18) [4]	107.3 (25.27) [15]
AIC								
C_max_, ng/mL					5.151 (28.12) [6]	4.431 (48.12) [16]	3.570 (122.10) [6]	3.298 (38.25) [18]
T_max_, h^a^					1.5 (1.0–1.5)	1.5 (1.0–3.0)	1.5 (1.5–3.0)	1.5 (1.5–3.0)
AUC_(0–8)_, ng•h/mL					21.54 (28.01) [5]	17.58 (46.87) [15]	15.05 (108.10) [6]	13.29 (38.39) [18]
AUC_(0–12)_, ng•h/mL					28.31 (19.91) [5]	22.32 (43.91) [7]	21.40 (90.82) [6]	19.38 (28.28) [11]

Docetaxel, 75 mg/m^2^; dacarbazine, 1000 mg/m^2^

^a^Median value and range

AIC, 5-aminoimidazole-4-carboxamide (main metabolite of dacarbazine); AUC_(0–8)_, area under the concentration-time curve from 0 to 8 h; AUC_(0–12)_, area under the concentration-time curve from 0 to 12 h; C_max_, maximum plasma concentration; T_max_, Time to reach maximum plasma concentration

#### Tumor response

**Fig. 2:**
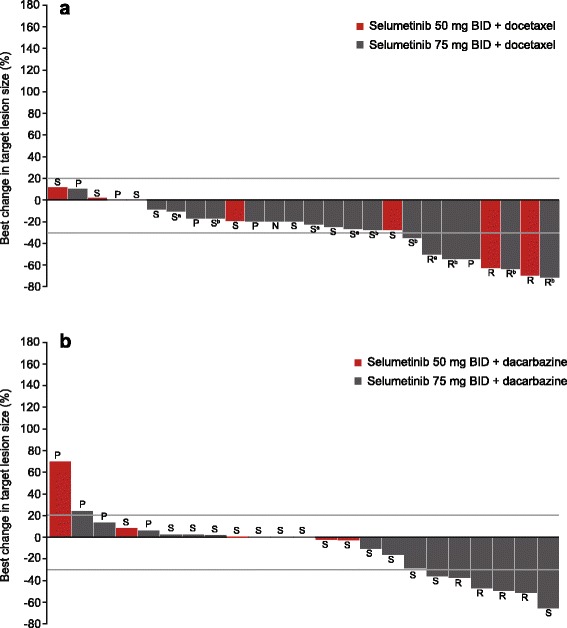
Waterfall plots for greatest change in target lesion size from baseline for the (**a**) selumetinib plus docetaxel and (**b**) selumetinib plus dacarbazine arms. Lower reference line indicates the point below which best response is partial response (>30% reduction). Upper reference line indicates the point above which best response is progressive disease (>20%). Administration of primary prophylactic granulocyte colony stimulating factor (ppG-CSF﻿) differed between the part A selumetinib 75 mg bid cohort due to differences in treatment practices between study centers: ^a^Received primary ppG-CSF﻿ in cycle 1; ^b^Did not receive ppG-CSF﻿ in cycle 1. Response Evaluation Criteria In Solid Tumors best response: N, not evaluable; P, progressive disease; R, partial response; S, stable disease. Population: Measurable disease at baseline and underwent follow-up scan

Six of 27 evaluable patients receiving selumetinib plus docetaxel had a confirmed partial response (melanoma, *n* = 2; lung, head and neck, ovarian, unknown primary, *n* = 1 each); *KRAS* or *EGFR* mutations were not detected in these patients. An additional 14 patients had a best response of stable disease ≥6 weeks, including two patients (one with lung cancer, one with bladder cancer) who had unconfirmed partial responses. Seven patients had progressive disease as best response. At week 12, 11 patients (31.4%) had stable disease and five patients (14.3%) had an objective response. The median duration of response in patients receiving selumetinib 75 mg BID plus docetaxel was 328 days (range 95 to 555). Greatest change from baseline (*n* = 26) in target lesion size is presented in Fig. 2.

### Selumetinib in combination with dacarbazine

#### Dose-limiting toxicities

Seven patients received selumetinib 50 mg BID plus dacarbazine, and one of six evaluable patients had a DLT (grade 4 thrombocytopenia). DLTs did not occur in the other selumetinib plus dacarbazine dose cohorts and selumetinib 75 mg BID plus dacarbazine was defined as the MTD in part A.

#### Exposure

For patients who received selumetinib 75 mg BID plus dacarbazine, the mean duration of selumetinib treatment was approximately 6 months, and 44% of patients had combination treatment for six or more (21-day) cycles. Two patients with lymph node or skin/soft tissue tumors who were ongoing at the time of data cut-off received selumetinib treatment for >18 months (2 years 9 months and 1 year 7 months, respectively) before discontinuing study treatment.

#### Tolerability

Diarrhea, peripheral/periorbital edema, nausea, and fatigue were the most commonly reported AEs among patients receiving selumetinib plus dacarbazine (Table 3). The most commonly reported grade ≥3 AEs were: anemia (4/25 patients, 16%), neutropenia (3/25, 12%), fatigue (3/25, 12%), and thrombocytopenia (2/25, 8%). With the exception of fatigue, non-hematologic grade ≥3 AEs were not reported in more than one patient.

Eleven patients (44%) experienced grade 1 AEs relating to vision disturbance. Seven patients (28%) reported blurred vision and four patients (16%) had vitreous floaters. AEs related to cardiac disorders were reported in two patients (8%) receiving selumetinib 75 mg BID; one patient (4%) had grade 2 left ventricular dysfunction that was considered related to selumetinib by the investigator and resolved, and one patient had grade 1 electrocardiogram QT prolongation, for which the investigator temporarily stopped treatment.

In part A, one patient each from the selumetinib 50 mg BID plus dacarbazine (14.3%) and selumetinib 75 mg BID plus dacarbazine (16.7%) arms required a dose reduction of selumetinib. One patient (8.3%) receiving selumetinib 75 mg BID plus dacarbazine in part B required a selumetinib dose reduction.

One patient with uterine cancer and lung and pleural metastases had grade 4 AEs of malignant pleural effusion and exertional dyspnea 34 days after starting treatment with selumetinib 50 mg BID plus dacarbazine. These events were considered by the investigator to be related to disease progression and not to study treatment. The primary cause of death was reported as fatal AEs of dyspnea exertional and malignant pleural effusion.

#### Pharmacokinetics analysis

Pharmacokinetic parameters of selumetinib, N-desmethyl selumetinib, dacarbazine, and 5-aminoimidazole-4-carboxamide (main metabolite of dacarbazine) were similar when administered alone and in combination (summarized in Table 4). Plasma concentration-time profiles of selumetinib, N-desmethyl selumetinib, and dacarbazine were also similar when administered alone or in combination (Additional file 3: Figure S2).

#### Tumor response

Four of 23 evaluable patients receiving selumetinib plus dacarbazine had a confirmed partial response (melanoma, *n* = 3; unknown primary cancer, *n* = 1) and 15 had a best response of stable disease ≥6 weeks (including one patient with a skin/soft tissue tumor and one with lung cancer who had unconfirmed partial responses). Six patients had progressive disease. None of the responding patients had primary tumor and/or plasma samples available for mutation analysis. At week 12, seven patients had stable disease, and two patients had an objective response. The median duration of response in patients receiving 75 mg BID plus dacarbazine was 245.5 (range 73 to 308) days. Greatest change from baseline in target lesion size is presented in Fig. 2.

## Discussion

At the time of study initiation, data from preclinical studies suggested that selumetinib in combination with a variety of DNA-damaging agents and molecularly targeted therapies may enhance anti-tumor efficacy compared with single agent administration [1, 11]. Combinations with docetaxel and dacarbazine have since shown clinical activity in phase II trials [12, 13].

This phase I study was the basis for establishing the safety, tolerability, MTD, and PK of selumetinib BID in combination with standard doses of two commonly used cytotoxic chemotherapeutic drugs in patients with advanced solid tumors. Data from patients who received selumetinib in combination with erlotinib or temsirolimus are presented in the companion paper (Infante et al., in preparation).

Selumetinib was tolerable in combination with docetaxel or dacarbazine. As with the monotherapy recommended phase II dose [4], we determined that the MTD of selumetinib was 75 mg BID when administered with standard doses of docetaxel 75 mg/m^2^ without ppG-CSF or with dacarbazine 1000 mg/m^2^. Consistent with our expectations, combination treatment reported here did not affect exposure to docetaxel, dacarbazine, or to selumetinib and its metabolite N-desmethyl selumetinib [6, 15, 16].

The tolerability of selumetinib in combination with docetaxel or dacarbazine was broadly consistent with the safety profiles of the individual regimen components [9, 17–19]. AEs that occurred most frequently with either combination were diarrhea, peripheral/periorbital edema, fatigue, nausea, and vomiting. Only a limited number of patients had ophthalmologic or LVEF assessments on study, so no definitive conclusions could be made relating to these domains.

The tolerability profiles in this phase I study have been seen in subsequent placebo-controlled, phase II trials of selumetinib plus dacarbazine in patients with treatment-naïve *BRAF-*mutant metastatic melanoma and selumetinib plus docetaxel in pretreated patients with *KRAS*-mutant advanced NSCLC [12, 13]. In both trials, the most frequently reported AEs for selumetinib combination arms were nausea, acneiform dermatitis, diarrhea, vomiting, and peripheral edema.

We determined a recommended phase II dose of selumetinib 75 mg BID plus docetaxel without ppG-CSF. However, in two phase II studies of selumetinib plus docetaxel without ppG-CSF for patients with treatment-naïve advanced melanoma and pretreated *KRAS*-mutant advanced NSCLC, increased incidences of grade ≥3 febrile neutropenia were reported compared with placebo (21% vs. 12% and 18% vs. 0%, respectively) [12, 20]. The reason for this discrepancy likely derives from the inaccuracy of many recommended phase II dose determinations based on studies of a relatively small number of carefully selected patients treated at phase I research centres. Two subsequent multicenter trials of selumetinib plus docetaxel for patients with advanced NSCLC included mandatory administration of ppG-CSF in the protocol: SELECT-1, evaluating the combination as second-line treatment in *KRAS*-mutant advanced NSCLC (ClinicalTrials.gov identifier: NCT01933932); and SELECT-2, evaluating the hypothesis that clinical activity of the combination is not limited to patients with advanced NSCLC that harbours *KRAS* mutations (ClinicalTrials.gov identifier: NCT01750281).

Although this study was not designed to assess clinical efficacy, objective tumor responses were reported in some patients. Among evaluable patients receiving selumetinib plus docetaxel, 6/27 (22%) had a confirmed partial response and an additional 14/27 (52%) had stable disease ≥6 weeks. At week 12, 11 (31%) patients had stable disease and five (14%) patients had an objective response. Interestingly, *KRAS* or *EGFR* mutations were not detected in any of the patients who responded, supporting the hypothesis under investigation in the SELECT-2 trial. Confirmed partial responses occurred in 4/23 (17%) evaluable patients receiving selumetinib plus dacarbazine and 15/23 (65%) had stable disease ≥6 weeks. At week 12, seven (28%) patients had stable disease, and two (8%) patients had an objective response.

## Conclusion

Selumetinib 75 mg BID in combination with dacarbazine or docetaxel (plus ppG-CSF as appropriate) demonstrated clinical activity and was reasonably well tolerated in patients with advanced solid tumors, informing further evaluation in randomized studies.

## Additional files

Additional file 1:
**Table S1.** Independent Ethics Committees/Institutional Review Boards consulted (DOCX 42 kb)
Additional file 2:
**Figure S1.** Plots of geometric mean (+/− standard deviation) plasma concentrations over time of (A) selumetinib 75 mg BID alone and in combination with docetaxel 75 mg/m^2^ or (B) docetaxel 75 mg/m^2^ alone and in combination with selumetinib 75 mg BID (EPS 1929 kb)
Additional file 3:
**Figure S2.** Plots of geometric mean (+/− standard deviation) plasma concentrations over time of (A) selumetinib 75 mg BID alone and in combination with dacarbazine 1000 mg/m^2^ or (B) dacarbazine 1000 mg/m^2^ alone and in combination with selumetinib 75 mg BID (EPS 1925 kb)

## Abbreviations

AE: Adverse event
AIC: 5-aminoimidazole-4-carboxamide
AUC_(0–12)_: Area under the plasma concentration-time curve from 0 to 12 h post dose
AUC_(0–8)_: Area under the plasma concentration-time curve from 0 to 8 h post dose
BID: Twice daily
CI: Confidence interval
C_max_: Maximum plasma concentration
CTCAE: National Cancer Institute Common Terminology Criteria for Adverse Events
CYP: Cytochrome
DLT: Dose-limiting toxicity
MTD: Maximum tolerated dose
NA: Not applicable
NSCLC: Non-small cell lung cancer
PK: Pharmacokinetics
RECIST: Response Evaluation Criteria In Solid Tumors
SAE: Serious adverse event
SD: Standard deviation
SRC: Safety review committee
T_max_: Time to reach maximum plasma concentration
WHO: World Health Organization
