# Supplementary material for: A phase I dose-escalation study of selumetinib in combination with docetaxel or dacarbazine in patients with advanced solid tumors
Source: BMC Cancer. 2017 Mar 6;17:173. doi: 10.1186/s12885-017-3143-6 (PMC5340007; doi:10.1186/s12885-017-3143-6)
Supplement: Additional file 1: — Table S1. Independent Ethics Committees/Institutional Review Boards consulted (DOCX 42 kb) [file 12885_2017_3143_MOESM1_ESM.docx]

# Additional files

**Table S1** Independent Ethics Committees/Institutional Review Boards consulted

| **Research site** | **Ethics committee** | **Committee number** |
| --- | --- | --- |
| Detroit, Michigan, USA | Wayne State University, Human Investigations Committee | 050508M1F |
| Austin, Texas, USA | IntegReview | IRB00001035, IRB00003657, IRB00004920, IRB00006075 |
| Rockledge, Pennsylvania, USA | Institutional Review Board, Fox Chase Cancer Canter | IRB #08-001 |
| Houston, Texas, USA | Institutional review Board, MD Anderson Cancer Center | IRB00000121 |

**Fig. S1** Plots of geometric mean (+/- standard deviation) plasma concentrations over time of (A) selumetinib 75 mg BID alone and in combination with docetaxel 75 mg/m^2^ or (B) docetaxel 75 mg/m^2^ alone and in combination with selumetinib 75 mg BID. The selumetinib plasma concentration data at 12 hours from one patient were excluded from the mean result as it appeared that the blood sample was taken after the second daily dose. BID, twice daily

**Fig. S2** Plots of geometric mean (+/- standard deviation) plasma concentrations over time of (A) selumetinib 75 mg BID alone and in combination with dacarbazine 1000 mg/m^2^ or (B) dacarbazine 1000 mg/m^2^ alone and in combination with selumetinib 75 mg BID. BID, twice daily

# 
